# Effectiveness of eHealth interventions for the promotion of physical activity in older adults: a systematic review protocol

**DOI:** 10.1186/s13643-016-0223-7

**Published:** 2016-03-16

**Authors:** Saskia Muellmann, Sarah Forberger, Tobias Möllers, Hajo Zeeb, Claudia R Pischke

**Affiliations:** Department Prevention and Evaluation, Leibniz Institute for Prevention Research and Epidemiology–BIPS, Achterstrasse 30, Bremen, 28215 Germany; Health Sciences Bremen, University of Bremen, Bremen, Germany

**Keywords:** Systematic review, Physical activity, Elderly, Adults, Healthy ageing, eHealth interventions, Primary prevention

## Abstract

**Background:**

It is known that regular physical activity (PA) is associated with improvements in physical, psychological, cognitive, and functional health outcomes. The World Health Organization recommends 150 min of moderate exercise per week for older adults to achieve these health benefits. However, only 20–60 % of adults aged 60 years and above currently meet these recommendations for exercise. The widespread use of the internet and mobile phones among older adults may open new opportunities to promote PA in this population. Findings of previous reviews suggest that eHealth interventions are effective in promoting PA in adults of various ages. However, to date, none of these reviews have provided a differentiated picture of engagement in such interventions and effects on PA among older adults. Also, we are unaware of any studies comparing effects of participation in eHealth vs. traditional paper-and-pencil interventions on PA in this population. The aim of this systematic review and meta-analysis is to compare the effectiveness of eHealth interventions promoting PA in older adults aged 55 years and above with either a non-eHealth PA intervention or a group that is not exposed to any intervention.

**Methods:**

Eight electronic databases (MEDLINE, EMBASE, CINAHL, CENTRAL, PEI, PsycINFO, Web of Science, and OpenGrey) will be searched to identify experimental and quasi-experimental studies examining the effectiveness of eHealth interventions for PA promotion in adults aged 55 years and above. Two authors will independently select and review references, extract data, and assess the quality of the included studies by using the Cochrane Collaboration’s risk of bias tool. Disagreements between authors will be resolved by discussion involving a third author. If feasible, a meta-analysis will be conducted. Narrative synthesis using harvest plots will be performed, should a meta-analysis not be feasible.

**Discussion:**

The proposed systematic review will be the first review that compares the effectiveness of eHealth interventions promoting PA in older adults aged 55 years and above with control groups exposed to a non-eHealth intervention or to no intervention. The results of this review will provide new information regarding the question whether eHealth interventions are an effective intervention vehicle for PA promotion in this population.

**Systematic review registration:**

PROSPERO CRD42015023875

**Electronic supplementary material:**

The online version of this article (doi:10.1186/s13643-016-0223-7) contains supplementary material, which is available to authorized users.

## Background

Regular physical activity (PA) and the reduction of sitting time or a predominantly sedentary lifestyle are associated with improvements in physical, psychological, cognitive, and functional health [1–3]. The World Health Organization (WHO) recommends a weekly moderate exercise time of 150 min per week for older adults to obtain health benefits [4]. Percentages of older adults aged 60 years and above meeting the recommended PA levels ranged from 2 - 83 %, depending on the study. In 45 of the 53 studies included in the systematic review by Sun and colleagues that reported PA levels for adult populations, 20 - 60 % of older adults met the recommendations [5].

Determinants of PA initiation and maintenance can be categorized into personal, psychological, social, and environmental factors. Personal determinants playing a role in the uptake and maintenance of PA include age (i.e., older adults become less active with increasing age), gender (i.e., men tend to be more active than women), and overall health status (i.e., good overall physical health is predictive of higher levels of PA) [6, 7]. Psychological and social determinants of PA include self-efficacy, perceived benefits, enjoyment, intention, and readiness to change behavior. Specifically, persons who report high levels of self-efficacy and have developed an intention (or readiness) to engage in the behavior, who perceive PA to be beneficial to their health, and who report enjoying PA tend to engage more frequently in the behavior. Determinants concerning the social or physical environment include the availability of a social network (i.e., having a good social network and/or a sports partner is associated with PA initiation and maintenance) and social support (e.g., receiving social support from significant others is associated with an initiation of PA), perceived safety of the environment, and perceived access to sports/exercise facilities [6]. Thus far, a multitude of studies addressing the above mentioned determinants of PA to varying degrees have demonstrated the effectiveness of PA interventions.

Furthermore, interventions providing information on PA as print versions or face-to-face have a long tradition [8–10]. However, the increased use of the internet and mobile technologies in recent years may open new opportunities to promote PA in adult populations, including older adults [11]. eHealth is defined as “the transfer of health resources and health care by electronic means” [12]. This includes, among other things, the delivery of health information through the internet and mobile technologies. In the elderly population, a growing number of individuals use electronic devices, such as computers, smartphones, or tablets [13]. Results of previous systematic reviews and meta-analyses indicate that eHealth interventions are an effective intervention vehicle to promote PA among adults of various ages [14–18]. However, in the majority of the studies included in these reviews, PA interventions were combined with other intervention components, such as recommendations for lifestyle changes, either for weight loss or the management of type 2 diabetes. Furthermore, the evidence for the effectiveness of these interventions in regard to PA promotion among older adults is mixed. While some studies suggest that eHealth approaches effectively promote PA [19–21], other studies report no beneficial effect of eHealth PA interventions compared to non-eHealth interventions [22, 23]. The aim of this systematic review and meta-analysis is to compare the effectiveness of eHealth interventions solely promoting PA in older adults aged ≥55 years with either a no intervention or a non-eHealth intervention. This systematic review protocol will adhere to the reporting guidelines of the Preferred Reporting Items for Systematic Reviews and Meta-Analyses Protocols (PRISMA-P) statement [24]. The completed PRISMA-P checklist can be found in the Additional file 1.

## Methods

### Study registration

This systematic review is registered at PROSPERO (registration number: CRD42015023875; http://www.crd.york.ac.uk/PROSPERO).

### Types of studies

Experimental (randomized controlled trial (RCT)) or quasi-experimental study designs that compare an eHealth PA intervention targeting older adults (≥55 years old) with either a non-eHealth PA intervention or a group that is not exposed to any intervention will be included.

### Types of participants

Studies including older adults of both sexes without severe pre-existing chronic medical conditions (e.g., cancer, coronary heart disease, cognitive impairment) aged ≥55 years will be included in our review.

### Types of interventions

Our review will include studies of eHealth interventions promoting PA in older adults. eHealth interventions encompass interventions accessible via computer, smartphone, or tablet. Studies will be included if the main intervention component is delivered via computer (i.e., website, e-mail, physical activity tracker) or smartphone/tablet (i.e., mobile app, text messaging, telephone calls, physical activity tracker). The interventions can be delivered to groups or individuals. Interventions can involve one-time or repeated online contacts with research and/or intervention teams. Intervention contacts can include counseling or advice or both, self-directed or prescribed exercise or both, home-based or facility-based exercise or both, and the provision of written education or motivational support materials or both. eHealth mass media interventions, DVD-based interventions, and interventions delivered using gaming consoles (e.g., Nintendo Wii) will be excluded.

### Types of comparators

Comparator conditions will include participation in (a) a non-eHealth intervention (e.g., paper-and-pencil intervention without eHealth component and face-to-face consultation, e.g., prescription of PA by a physician or exercise in groups or with personal trainer) or (b) no intervention.

### Types of outcomes

PA can be assessed using objective (e.g., pedometer, accelerometer) or subjective methods (e.g., PA diary, questionnaires).

## Search methods for the identification of studies

### Data collection and analyses

The following databases will be searched:

- ➢ MEDLINE (via PubMed, 1946 to present),
- ➢ PsycINFO (via Ovid, 1806 to present),
- ➢ Web of Science including Social Sciences Citation Index and Science Citation Index Expanded (1900 to present),
- ➢ Cumulative Index to Nursing & Allied Health Literature (CINAHL) (via EBSCO Host, 1981 to present),
- ➢ Excerpta Medica Database (EMBASE) (via Ovid, 1974 to present),
- ➢ Cochrane Central Register of Controlled Trials (CENTRAL) (via Cochrane Library, 1948 to present),
- ➢ Physical Education Index (PEI) (via ProQuest, 1970 to present),
- ➢ and OpenGrey (1980 to present).

The search is restricted to studies published in English or German. Keywords will be related to PA, older adults, and eHealth interventions, using MeSH terms and other index terms, as well as appropriate synonyms. The keywords will be combined using the Boolean operation OR and AND. Validated RCT filters will be used for the searches in MEDLINE, PsycINFO, Web of Science, CINAHL, and EMBASE. For PEI and OpenGrey, no validated RCT filters are available. Therefore, appropriate keywords to identify studies using an experimental or quasi-experimental study design will be employed. For the search in CENTRAL, no RCT filter is necessary because the database only includes controlled trials. The search strategy is illustrated in Additional file 2 using the MEDLINE search as an example. References of the included studies will be searched to identify additional potentially relevant studies.

### Selection of studies

One author will conduct the database search. First, titles and abstracts of studies identified using the search strategy outlined above will be screened independently by two authors to select the relevant studies. Any disagreements between the two authors will be discussed until consensus is reached. A third author will be involved in this discussion and will facilitate the process when necessary. In a second step, full texts of potentially relevant studies will be obtained and reviewed independently by two authors. Any disagreements between the two authors will be resolved by consensus and/or discussion with a third author. Screening of titles, abstracts, and full texts will be performed by uploading citations to the online systematic review software “Covidence” by Alfred Health.

### Data extraction

Data extraction will be conducted independently by two authors. In case of disagreements that cannot be resolved by discussion, a third author will be involved. The following information will be extracted from the included studies: publication details (first author, year, title, country of study, funding source), study design, information regarding study methods (aim of study, inclusion/exclusion criteria for study participation, recruitment of participants, randomization procedure, statistical analyses, study limitations), participant characteristics (number of participants, number of withdrawals/excluded participants, mean/median age, age range, sex, ethnicity, existing risk factors for chronic diseases (e.g., type 2 diabetes)), intervention details (name of intervention, aim of intervention, number of intervention/control groups, intervention components and levels, delivery of intervention, intervention setting, duration of intervention, duration of follow-up, underlying theory), primary and secondary outcomes, outcome measurement, time points when data were collected, intervention effects on primary and secondary outcomes, and authors’ conclusions.

### Assessment of risk of bias in included studies

Two authors will independently assess the risk of bias in the included studies by using the Cochrane Collaboration’s risk of bias tool [25]. Potential disagreements will be resolved as outlined above.

### Data synthesis

A narrative synthesis of all relevant studies will be provided with tables of study and participants’ characteristics, details regarding intervention components and levels, outcomes, results, and authors’ conclusions. If feasible, a quantitative synthesis (i.e., meta-analysis) will be performed by using the random effects model because it is expected that the included studies will be heterogeneous in terms of intervention components and levels, physical activity assessment, and the comparator groups (i.e., non-eHealth intervention, no intervention). Heterogeneity will be assessed by visual inspection and calculating the *I*^2^ statistics [26]. Sensitivity analysis based on study quality and study design will be performed to determine the robustness of our results (i.e., studies considered as “low risk of bias” compared with studies considered as “high risk of bias”, RCTs compared with quasi-experimental studies). If a meta-analysis is not feasible, narrative synthesis for summarizing the evidence with regard to intervention effects, using harvest plots, will be performed [27].

### Subgroup analyses

If the selected data permit, a narrative and quantitative synthesis will be provided for the following subgroups and subsets of studies: subjective or objective assessment of PA, intervention access (computer, smartphone, or tablet), and intervention components and levels (e.g., information regarding recommendations for PA and benefits of regular PA, skills training/instructions, social comparison/social support, individual level vs. interventions incorporating or targeting features of the social or physical context).

## Discussion

This systematic review will be performed to compare the effectiveness of eHealth interventions for the promotion of PA in older adults aged ≥55 years with non-eHealth interventions or no exposure to any interventions. This review is important because many European countries are currently facing significant demographic change and population-based strategies to promote PA which are needed to improve older adults’ health, in general, and to prevent frailty and/or the progression of chronic disease risk factors to chronic diseases which pose a significant burden on European Union health-care systems. A major advantage of eHealth interventions is that such interventions are easily accessible and usable (possibly only with little assistance of intervention or research teams) and that segments of populations can be reached by who may not otherwise get in contact with traditional health promotion or PA interventions [28]. Once these interventions are developed and adequately tested and tailored to individual needs of older adults, technology-based interventions may be more cost-effective than person-based interventions for PA promotion.

To our knowledge, this will be the first review that synthesizes information on the effectiveness of eHealth interventions promoting PA in older adults aged 55 years and above. We also hope to find evidence regarding the impact of different intervention components and the role of social or environmental changes incorporated in interventions addressing individual PA behavior. However, we expect that the studies identified for this review will be very heterogeneous in terms of intervention components and levels, health outcomes assessed, and the number of comparator groups. Should we find a sufficient number of studies with comparable interventions and similar assessments of health outcomes, we aim to synthesize the evidence in a meta-analysis. We are also aware that our findings will depend on the number of eligible studies identified and the quality of these studies. Also, eight databases which are relevant to our topic will be searched, but it is possible that not all relevant studies are included in these databases. Furthermore, assuming that the core body of evidence will be covered, our search will be restricted to studies published in English and German.

## Additional files

Additional file 1: PRISMA-P 2015 checklist: recommended items to address in a systematic review protocol. (PDF 212 kb) Additional file 2: MEDLINE search. (PDF 193 kb)

## Abbreviations

CENTRAL: Cochrane Central Register of Controlled Trials
CINAHL: Cumulative Index to Nursing & Allied Health Literature
EMBASE: Excerpta Medica Database
PA: physical activity
PEI: Physical Education Index
PRISMA-P: Preferred Reporting Items for Systematic Reviews and Meta-Analyses Protocols
RCT: randomized controlled trial
WHO: World Health Organization
