# Supplementary material for: Effectiveness of eHealth interventions for the promotion of physical activity in older adults: a systematic review protocol
Source: Syst Rev. 2016 Mar 16;5:47. doi: 10.1186/s13643-016-0223-7 (PMC4793489; doi:10.1186/s13643-016-0223-7)
Supplement: Additional file 1: — PRISMA-P 2015 checklist: recommended items to address in a systematic review protocol. (PDF 212 kb) [file 13643_2016_223_MOESM1_ESM.pdf]

Additional file 1: PRISMA-P 2015 checklist: recommended items to address in a systematic review protocol [24].

Section and topic	Item No	Checklist item
**Administrative information**		
Title		
Identification	1a	Identify the report as a protocol of a systematic review **(Title)**
Update	1b	If the protocol is for an update of a previous systematic review, identify as such **(Not applicable)**
Registration	2	If registered, provide the name of the registry (such as PROSPERO) and registration number **(Abstract)**
Authors		
Contact	3a	Provide name, institutional affiliation, e-mail address of all protocol authors; provide physical mailing address of corresponding author **(Title page)**
Contributions	3b	Describe contributions of protocol authors and identify the guarantor of the review **(Authors' contributions)**
Amendments	4	If the protocol represents an amendment of a previously completed or published protocol, identify as such and list changes; otherwise, state plan for documenting important protocol amendments **(Not applicable)**
Support		
Sources	5a	Indicate sources of financial or other support for the review **(Funding information)**
Sponsor	5b	Provide name for the review funder and/or sponsor **(Funding information)**
Role of sponsor or funder	5c	Describe roles of funder(s), sponsor(s), and/or institution(s), if any, in developing the protocol **(Not applicable)**
**Introduction**		
Rationale	6	Describe the rationale for the review in the context of what is already known **(Background)**
Objectives	7	Provide an explicit statement of the question(s) the review will address with reference to participants, interventions, comparators, and outcomes (PICO) **(Background, methods: types of studies/ participants/ interventions/ comparators/ outcomes)**
**Methods**		
Eligibility criteria	8	Specify the study characteristics (such as PICO, study design, setting, time frame) and report characteristics (such as years considered, language, publication status) to be used as criteria for eligibility for the review **(Methods: types of studies/ participants/ interventions/ comparators/ outcomes, data collection and analyses)**
Information sources	9	Describe all intended information sources (such as electronic databases, contact with study authors, trial registers or other grey literature sources) with planned dates of coverage **(Methods: data collection and analyses)**

Search strategy	10	Present draft of search strategy to be used for at least one electronic database, including planned limits, such that it could be repeated **(Additional file 2)**
Study records		
Data management	11a	Describe the mechanism(s) that will be used to manage records and data throughout the review **(Methods: selection of studies)**
Selection process	11b	State the process that will be used for selecting studies (such as two independent reviewers) through each phase of the review (that is, screening, eligibility and inclusion in meta-analysis) **(Methods: selection of studies, data extraction, assessment of risk of bias in included studies)**
Data collection process	11c	Describe planned method of extracting data from reports (such as piloting forms, done independently, in duplicate), any processes for obtaining and confirming data from investigators **(Methods: data extraction)**
Data items	12	List and define all variables for which data will be sought (such as PICO items, funding sources), any pre-planned data assumptions and simplifications **(Methods: data extraction)**
Outcomes and prioritization	13	List and define all outcomes for which data will be sought, including prioritization of main and additional outcomes, with rationale **(Methods: types of outcomes)**
Risk of bias in individual studies	14	Describe anticipated methods for assessing risk of bias of individual studies, including whether this will be done at the outcome or study level, or both; state how this information will be used in data synthesis **(Methods: assessment of risk of bias in included studies)**
Data synthesis	15a	Describe criteria under which study data will be quantitatively synthesised **(Methods: data synthesis)**
	15b	If data are appropriate for quantitative synthesis, describe planned summary measures, methods of handling data and methods of combining data from studies, including any planned exploration of consistency (such as I^2, Kendall's τ) **(Methods: data synthesis)**
	15c	Describe any proposed additional analyses (such as sensitivity or subgroup analyses, meta-regression) **(Methods: data synthesis, subgroup analyses)**
	15d	If quantitative synthesis is not appropriate, describe the type of summary planned **(Methods: data synthesis)**
Meta-bias(es)	16	Specify any planned assessment of meta-bias(es) (such as publication bias across studies, selective reporting within studies) **(Not applicable)**
Confidence in cumulative evidence	17	Describe how the strength of the body of evidence will be assessed (such as GRADE) **(Not applicable)**
