# Supplementary material for: Effectiveness of eHealth interventions for the promotion of physical activity in older adults: a systematic review protocol
Source: Syst Rev. 2016 Mar 16;5:47. doi: 10.1186/s13643-016-0223-7 (PMC4793489; doi:10.1186/s13643-016-0223-7)
Supplement: Additional file 2: — MEDLINE search. (PDF 193 kb) [file 13643_2016_223_MOESM2_ESM.pdf]

Additional file 2: Medline search.

Medline search (via PubMed, 30th October 2015)

Physical activity

Search name	Search query	Type of search	Results
#1	physical education and training[MeSH Terms] OR sports[MeSH Terms] OR exercise[MeSH Terms] OR physical fitness[MeSH Terms] OR exercise therapy[MeSH Terms] OR motor activity[MeSH Terms]	MeSH terms	305,405
#2	sedentary behavio*[Title/Abstract] OR physical activi*[Title/Abstract] OR physical inactivi*[Title/Abstract] OR sport*[Title/Abstract] OR exercis*[Title/Abstract] OR muscle stretching exercise*[Title/Abstract] OR resistance training[Title/Abstract] OR walk*[Title/Abstract] OR bicycle*[Title/Abstract] OR cycling[Title/Abstract] OR swim*[Title/Abstract] OR running*[Title/Abstract] OR gymnastic*[Title/Abstract] OR yoga[Title/Abstract] OR dancing[Title/Abstract] OR pilates[Title/Abstract] OR gardening[Title/Abstract]	Keyword TI/AB	480,553
#3	#1 OR #2		619,588

EHealth

Search name	Search query	Type of search	Results
#4	telemedicine[MeSH Terms] OR computer-assisted instruction[MeSH Terms] OR multimedia[MeSH Terms] OR computer systems[MeSH Terms] OR computers[MeSH Terms] OR cd-rom[MeSH Terms] OR electronic mail[MeSH Terms] OR cell phones[MeSH Terms] OR mobile applications[MeSH Terms] OR internet[MeSH Terms]	MeSH terms	168,712
#5	compute*[Title/Abstract] OR web*[Title/Abstract] OR online[Title/Abstract]	Keyword TI/AB	620,413
#6	#4 OR #5		718,731

Older adults

Search name	Search query	Type of search	Results
#7	aged[MeSH Terms]	MeSH terms	2,460,407
#8	elder*[Title/Abstract] OR older people[Title/Abstract] OR older adult*[Title/Abstract] OR old adult*[Title/Abstract] OR	Keyword TI/AB	295,730

	older person*[Title/Abstract] OR old person*[Title/Abstract] OR aging adult*[Title/Abstract] OR aging person*[Title/Abstract] OR ageing adult*[Title/Abstract] OR ageing person*[Title/Abstract] OR geriatrics[Title/Abstract] OR senior*[Title/Abstract]		
#9	#7 OR #8		2,561,541

RCT-Filter: sensitivity-maximizing version [26]

Search name	Search query	Results
#10	randomized controlled trial[pt]	398,286
#11	controlled clinical trial[pt]	89,554
#12	randomized[tiab]	358,994
#13	placebo[tiab]	170,382
#14	drug therapy[sh]	1,790,185
#15	randomly[tiab]	241,572
#16	trial[tiab]	409,061
#17	groups[tiab]	1,538,383
#18	#10 OR #11 OR #12 OR #13 OR #14 OR #15 OR #16 OR #17	3,679,368
#19	animals[mh] NOT humans[mh]	4,056,964
#20	#18 NOT #19	3,172,158

Summary and results

Search name	Search query	Results
#21	#3 AND #6 AND #9	4,729
#22	#3 AND #6 AND #9 AND #20	1,703
#23	#3 AND #6 AND #9 AND #20 Filters: English, German	1,622
